# Molecular Dynamic Simulations Reveal the Activation Mechanisms of Oxidation-Induced TRPV1

**DOI:** 10.3390/ijms24119553

**Published:** 2023-05-31

**Authors:** Yanyan Chu, Huanhuan Zhang, Mengke Yang, Rilei Yu

**Affiliations:** 1 Marine Biomedical Research Institute of Qingdao, Ocean University of China, 5 Yushan Road, Qingdao 266003, China; 2 Innovation Platform of Marine Drug Screening & Evaluation, Pilot National Laboratory for Marine Science and Technology (Qingdao), Qingdao 266100, China

**Keywords:** TRPV1, ion channel, allosteric mechanism, accelerated molecular dynamics, disulfide bond

## Abstract

Transient receptor potential vanilloid 1 (TRPV1), a non-selective cation channel, can be directly activated by oxidants through cysteine modification. However, the patterns of cysteine modification are unclear. Structural analysis showed that the free sulfhydryl groups of residue pairs C387 and C391 were potentially oxidized to form a disulfide bond, which is expected to be closely related to the redox sensing of TRPV1. To investigate if and how the redox states of C387 and C391 activate TRPV1, homology modeling and accelerated molecular dynamic simulations were performed. The simulation revealed the conformational transfer during the opening or closing of the channel. The formation of a disulfide bond between C387 and C391 leads to the motion of pre-S1, which further propagates conformational change to TRP, S6, and the pore helix from near to far. Residues D389, K426, E685–Q691, T642, and T671 contribute to the hydrogen bond transfer and play essential roles in the opening of the channel. The reduced TRPV1 was inactivated mainly by stabilizing the closed conformation. Our study elucidated the redox state of C387–C391 mediated long-range allostery of TRPV1, which provided new insights into the activation mechanism of TRPV1 and is crucial for making significant advances in the treatment of human diseases.

## 1. Introduction

Reactive oxygen species (ROS) are associated with pathological pain, including neuropathic pain, inflammatory pain, and visceral pain [1,2]. Redox-sensitive ion channels, which change membrane potential and intracellular ionic content by rapid activation kinetics, play an important role in sensing acute changes in the redox status. Among these channels, the transient receptor potential (TRP) channels have emerged as acute sensors of redox status and play an essential role in signal transduction by rapidly converting changes in the redox state to Na^+^ and Ca^2+^ influx [3,4]. 

The TRP channels are divided into six subfamilies, including vanilloids (TRPV), ankyrin (TRPA), canonical (TRPC), melastatins (TRPM), mucolipins (TRPML), and polycystins (TRPP) [5,6]. TRPM2, TRPM7, TRPC5, TRPV1, and TRPA1 are sensitive to various redox-regulating compounds, such as hydrogen peroxide (H_2_O_2_), nitric oxide (NO), and electrophiles [3,7]. Among these, the polymodal receptor TRPV1 is a non-selective cation channel that is activated by a wide range of chemical and physical stimuli, including heat (>43 °C), acidic pH, force, and numerous endogenous and exogenous ligands [8,9]. Many cellular signaling pathways regulate TRPV1 to alter the excitability of nociceptive neurons. TRPV1 is associated with numerous biological processes, such as autoimmune disorders, cancer, and immune cell function [10,11,12,13]. Considering that tissue damage and inflammation produce ROS, sensitization of TRPV1 under oxidative challenge is likely to play a role in nociceptor pain sensation during inflammation, infection, and tissue injury [14,15]. TRPV1 is sensitized and even activated by UVA-light, which is mainly mediated by the cysteine-dependent redox sensitivity of TRPV1 [16]. Most documented modifications caused by ROS are due to the presence of redox-sensitive cysteine thiol groups in proteins [17]. Exploring which cysteines are associated with TRPV1 activation and investigation. The activation mechanism is crucial for significant advances in the treatment of human diseases. 

**Figure 1:**
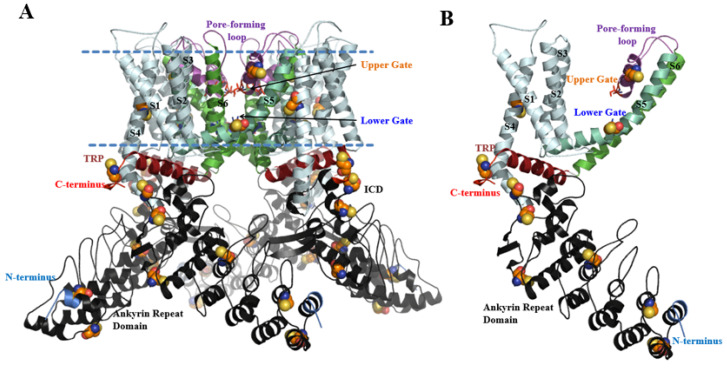
The structural overview of TRPV1 (PDB: 3J5Q [17]). The key structural features were labeled and colored differently. The region between the two dashed lines is transmembrane. (**A**) The whole structure of TRPV1 tetramer; (**B**) The monomer of TRPV1.

The structure of rat TRPV1 in both open and closed conformational states has been determined by cryo-electron microscopy (cryo-EM) [18,19,20]. TRPV1 is a homo-tetramer (Figure 1). Each TRPV1 subunit consists of six transmembrane α-helices (S1–S6) and an intracellular domain (ICD) [18,19,20]. The S1–S4 domain is located at the periphery of the channel. The S5–S6 domains from all four subunits surround a central pore formed by the extracellular linker located between S5–S6 [19]. The C- and N-terminus are located in the intracellular domain. The N-terminus of ICD forms an ankyrin repeat domain (ARD) and is involved in protein-protein interactions with components of the receptor signalplex [21,22]. The C-terminus of the ICD harbors a TRP domain, which is a conserved α-helix [23]. TRPV1 contains two gatekeepers: the upper gate G643–M644 of TRPV1 in the selectivity filter near the outer pore and the lower gate I679 of TRPV1 in S6, respectively [24,25]. The TRP helix interacts with the S4–S5 linker and is involved in the correct coupling of the activation stimuli and gate opening [23,26].

TRP channels utilize cysteine residues to sense alterations in redox status [3,27,28,29]. Cysteine oxidation has been considered the principal mechanism of TRPV1 oxidation sensing [7,30]. The human TRPV1 contains 16 cysteines according to its sequence. Several extracellular and intracellular cysteine residues can be oxidized to activate the TRPV1 channel and are responsible for redox sensing [4,28,29,31]. A site-specific mutation of cysteine indicated that the cytoplasmic cysteine residues located in the C-terminal ARD sensitize TRPV1 activation upon oxidative challenge. The homologous protein TRPA1 is also activated by electrophiles following covalent modification of cysteine residues localized within the ARD [3,32]. It was reported that the pungent compound allicin activates TRPV1 through covalent modification of C157 [4,30]. By analyzing all the determined structures of TRPV1, we noted that the cysteine pairs C386 and C390 are spatially close to each other, potentially forming a disulfide bond. However, the roles of the cysteine pair C386–C390 have never been studied. Considering that TRPV1 contains multiple activation mechanisms and that the mechanisms of human TRPV1 have been less studied, in the present study, we aimed to closely examine if and how the redox states of C386–C390 activate hTRPV1 and to evaluate the redox sensitivity of TRPV1. By using homology modeling and long-distance accelerated molecular dynamic simulations, we investigated the role of the cysteine pair in human TRPV1 activation by comparing the formation or breakage of a disulfide bond between C387 and C391 (corresponding to C386 and C390 in rat TRPV1). The TRPV1 channel opening from the closed state was observed when the cysteine pair C387 and C391 formed a disulfide bond, and the conformational changes of the subdomains related to conformational transfer were described in detail. The formation or not of disulfide bonds was expected to reflect, to some extent, the ROS situation of the physiological environment. The results reported here provide new insights into the activation mechanisms of human TRPV1 channels, which may be associated with ROS activation of TRPV1. Moreover, the results provided a structural basis to guide future functional studies of TRPV1.

## 2. Results and Discussions

Cysteine oxidation has been considered the principal mechanism of TRPV1-mediated alterations in redox status [7,30]. The cysteine pairs C387 and C391 of human TRPV1 (C386 and C390 in rat TRPV1) were reported to form an intra-subunit disulfide bond by Chuang [28]. However, the structure of the full-length human TRPV1 has not been resolved. Most of the cryo-EM structures of rat TRPV1 showed that the distance between the two residues is beyond the range of disulfide bond formation. After carefully analyzing all TRPV1 structures, we observed a structure with spatial accessibility of C386 and C390 (PDB: 5IRZ [18]), which can form a disulfide bond, even though the electron density map of their side chains is not clear [18]. Recently, Kwon [33] obtained a set of TRPV1 structures, PDB ID: 7RQW, 7RQV, 7RQU, 7RQX, 7RQY, and 7RQZ, whose corresponding cysteine pair was able to form a disulfide bond. The distance between the two sulfur atoms of the cysteine pair varies from 2.03 to 2.53. It is evident that the two cysteines exist in two possible states: the oxidized state and the reduced state. This kind of cysteine pair is also found in the homologous protein TRPV2, where C195 and C206 form a disulfide bond in the X-ray structure (PDB: 2ETC [34]), while the disulfide bond is broken in another X-ray structure (PDB: 2ETB [34]). To investigate the role of the cysteine pair in TRPV1 activation, we constructed the structures of human TRPV1 in different redox states. 

The cTRPV1 and oTRPV1 models, L112 to A720, were constructed by multiple template-based homology modeling (see supporting information, Figures S1 and S2) with and without the disulfide bond between the cysteine pair C387 and C391 manually. Therefore, four systems were obtained. The cTRPV1 with a disulfide bond was named cTRPV1-oxi, and the cTRPV1 without disulfide bonds was named cTRPV1-red. Accordingly, the oTRPV1 with or without a disulfide bond was named oTRPV1-oxi or oTRPV1-red, respectively. The MolProbity [35] server was used to evaluate the accuracy of the models. The MolProbity scores (number of overlaps > 0.4 Å per thousand atoms) of the constructed structures were 3.01 for cTRPV1-oxi, 3.06 for cTRPV1-red, 3.71 for oTRPV1-oxi, and 3.78 for oTRPV1-red, corresponding to the 98th percentile, 98th percentile, 96th percentile, and 96th percentile (N = 1784, all resolutions), respectively. The results suggest that the quality of the generated models is excellent.

### 2.1. Equilibrium MD Simulations

In addition, to assess the stability of TRPV1 during MD simulations, we calculated the backbone RMSD of the TRPV1 transmembrane domain together with the TRP helix, including residues 432–713. All systems remained stable after 100 ns, with RMSD values fluctuating in the range from 1.5 to 2.3 Å (Figure S3). Most disulfide bonds serve to stabilize protein structure. The larger the number of residues between the disulfide, the greater the stability imparted to the native structure [36]. Since there are only three residues between C387 and C391, the disulfide bond did not improve the overall stability of the system.

**Figure 2:**
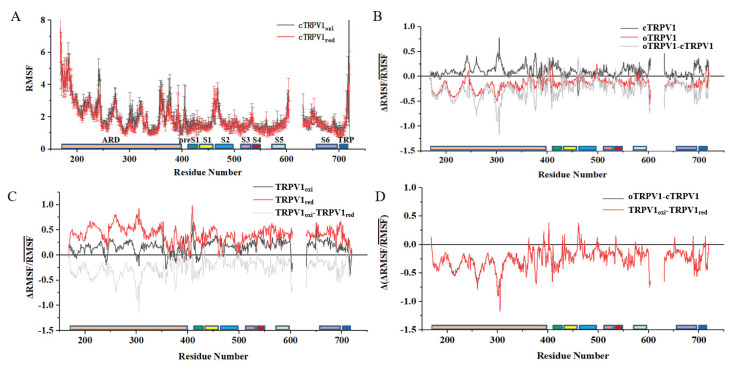
The RMSF and $\Delta RMSF/\bar{\mathrm{RMSF}}$ profiles for representative MD trajectories. The residue positions corresponding to the ARD, preS1, S1, S2, S3, S4, S5, S6, and TRP helix are labeled and colored in the same color. (**A**) The RMSF values of each residue in cTRPV1-oxi (black line) and cTRPV1-red (red line) systems; (**B**) The $\Delta RMSF/\bar{\mathrm{RMSF}}$ profiles for cTRPV1 (black line) and oTRPV1 (red line). The gray line stands for the difference between red line and black line; (**C**)The $\Delta RMSF/\bar{\mathrm{RMSF}}$ profiles between the open and closed TRPV1 systems with C387-C391 forming a disulfide bond (TRPV1oxi, black line), or with the reduced cysteine pair (TRPV1red, red line). The gray line stands for the difference between red line and black line; (**D**) The gray lines from figure (**B**) (black) and (**C**) (red) overlaid well.

Further, to assess the conformational flexibility at individual residue positions, we calculated the RMSF for each residue in the four systems (Figure 2A and Figure S4). Overall, the RMSF profiles show that the transmembrane helixes are rigid, with RMSF values below 2 Å. Several sharp peaks exhibit in the ARD, especially the N-terminal of the ARD, in the MPD linker connecting the ARD and S1 helix (Ser405 and E406), the C terminus of the TRP helix (C716–A720), and linkers connecting transmembrane helixes, such as the S1–S2 linker (near E467–T469) and the outer pore loop of the S5–S6 linker (near K603 and L631) (Figure 2A). Comparing the RMSD values in different redox states or in different subunits, no significant differences were found. The observed flexible residues are highly consistent with the previous report on TRPV1 heat activation [24,25]. 

To evaluate the effect of redox on RMSF, we calculated the fractional change in RMSF ($\Delta RMSF/\bar{\mathrm{RMSF}}$) for both oTRPV1 and cTRPV1 using function (1), where ${\mathrm{RMSF}}_{1}$ is the RMSF value in the oxidation states and ${\mathrm{RMSF}}_{2}$ is the RMSF in the reduction state (Figure 2B). Overall, $\Delta RMSF/\bar{\mathrm{RMSF}}$ values of cTRPV1 are essentially above zero, while values of oTRPV1 are almost below zero. The results indicate that residues of cTRPV1 is more flexible in the oxidation state than in the reduction state, while residues of oTRPV1 are less flexible in the oxidation state compared with the reduction state. For cTRPV1, the $\Delta RMSF/\bar{\mathrm{RMSF}}$ values are mainly in the range of 0 to 0.4, and there are several sharp peaks in ARD, the MPD linker (N409, H411, D412), the pre-S1 (R421), the S1-S2 linker (L461, P462), and the S4-S5 linker (M562, E570, K571, R575). For oTRPV1, the $\Delta RMSF/\bar{\mathrm{RMSF}}$ values range from −0.5 to 0.3, and several broad peaks exhibit in ARD, including the MPD linker (N409, V416), the S4–S5 linker (I564), and S6 (N677, M683). The observed flexible regions are partly consistent with the previous reports on TRPV1 conformational dynamics varying with temperature [25,37]. It suggests that the redox status of the cysteine pair may be related to TRPV1 activation by a mechanism similar to its thermal activation by affecting the flexibility of the same TRPV1 residues. The MPD linker and S4–S5 linker adjacent to the transmembrane domain play key roles in TRPV1 activation, while ARD at the distal end of the transmembrane domain may also have effects on TRPV1 activation via conformational transfer. The pre-S1, S1–S2 linker, and S6 regions are also more or less involved in activation, probably through fine-tuning the transmembrane region or being regulated by the transmembrane region during channel opening. These RMSF results were highly consistent with those observed in the study of heat activation mechanisms [25]. 

The $\Delta RMSF/\bar{\mathrm{RMSF}}$ profiles for TRPV1 in the same redox status and different conformations were calculated, where ${\mathrm{RMSF}}_{1}$ is the RMSF value of oTRPV1 and ${\mathrm{RMSF}}_{2}$ is the RMSF of cTRPV1 (Figure 2C). The obvious difference occurs in the ARD, pre-S1, S4-S5 linker, TRP, and S6-TRP linker, which is highly consistent with the above observations. Interestingly, the $\Delta RMSF/\bar{\mathrm{RMSF}}$ difference between oTRPV1 and cTRPV1 overlapped well with the difference between oxidized TRPV1 and reduced TRPV1 (Figure 2D). The results demonstrate that the redox status of C387–C391 is closely related to TRPV1 activation.

### 2.2. REDOX Regulates the Size of TRPV1 Channel

**Figure 3:**
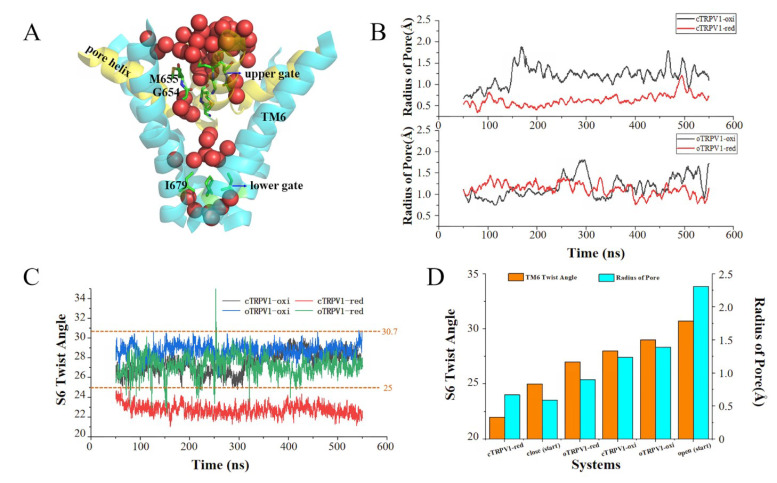
Characterization of the pore and S6 twist angle profiles. (**A**) The distribution of the water molecules in the pore of TRPV1; (**B**) The radius of TRPV1 pore profiles for representative MD trajectories, unit: Å; (**C**) The curve of S6 twist angles with simulation time; (**D**) The average S6 twist angle and the average radius of pore after simulation.

To monitor the movement of the channel, 2000 snapshots were extracted from the last 500 ns aMD trajectories of each system, and the minimal pore radius of each snapshot was measured using the Hole2 program [38]. The minimal pore radius of the starting structures was 0.59 Å for cTRPV1 and 2.31 Å for oTRPV1, respectively. The upper gate and the lower gate of the starting structures are closed or open simultaneously, and the radius of the upper gate is smaller than the lower gate (Figure S5). To facilitate analysis, the pore radius with running time was plotted (Figure 3). Obvious changes in pore radius were observed to be related to the redox status of the cysteines. 

In the oxidized state, both the oTRPV1-oxi and cTRPV1-oxi systems were closed after the initial 50 ns conventional MD simulations, with pore radii about or below 1 Å, and there are two broad peaks in each pore radius curve with values above 1.5 Å (Figure 3B). The radius of water has been reported to be 1.5 Å, the radius of Ca^2+^ is 0.99 Å [39], and the radius of a hydrated calcium ion is 1.34 Å [40]. Recently, Rao et al. [41] quantified the combined influence of radius and hydrophobicity on pore dewetting and showed that the hydrophobic gate opens with a minimum radius of about 2.0 Å. Thus, in the oxidized state, the conformation of TRPV1 switches freely between the closed and intermediate sub-states [33]. During the dynamic process, about 60% of snapshots are water permeable (with a pore radius > 1.15 Å [37]). 

In the reduced state, both cTRPV1-red and oTRPV1-red are closed and remain in a stable closed conformation during aMD, with an average pore radius of 0.67~0.9 Å, and only 10~20% of snapshots being water permeable.

Therefore, the redox status of the cysteine pairs C387 and C391 plays essential roles in TRPV1 activation and deactivation. The reducing state inactivates TRPV1 by stabilizing its closed conformation. The oxidizing state may play a role in TRPV1 (partial) activation by leading the channel to an intermediate state. This state was previously captured by cryo-EM [33] and has been observed in the TRPV2-Quad channel through a mechanism bridging the S4–S5 linker to the S1–S4 domain [42]. 

As an important part of the channel, the conformational change of S6 is directly related to the switching of TRPV1. The twist angle of S6 was measured during the MD simulations (Figure 3C,D). The S6 twist angle for the starting structure was 25° for closed TRPV1 and 30.7° for open TRPV1. In the reduced state, the S6 twist angles of cTRPV1-red decreased slightly to 22° during the simulations, and the angle of oTRPV1-red fluctuated significantly at the first 350 ns and then tended to be stable at an average of 27°. In the oxidized state, the S6 twist angle of cTRPV1-oxi increased to 28°, and the angle of oTRPV1-oxi is the largest at an average of 29°. Therefore, the S6 twist angles in ascending order are cTRPV1-red, the starting closed TRPV1, oTRPV1-red, cTRPV1-oxi, oTRPV1-oxi, and the starting open TRPV1 (Figure 3D). This trend coincides well with the trend in pore size. 

### 2.3. PCA Analysis

**Figure 4:**
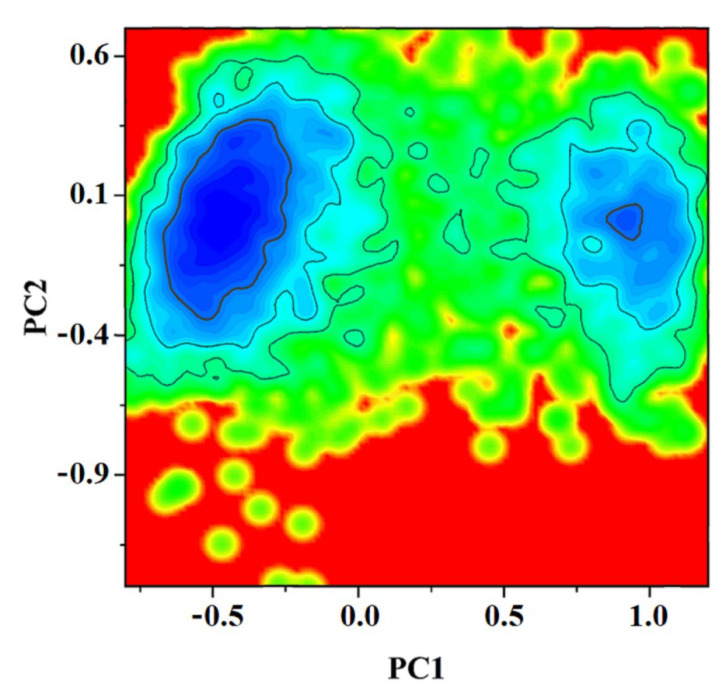
The principal component (PC) analysis of cTRPV1-oxi. From blue to red indicates the free energy from the lowest to the highest.

To analyze and visualize the overall motions of TRPV1 from the closed state to the intermediate state, dihedral covariance matrix calculation and projection, also called principal component analysis (PCA), were performed. PCA extracts the correlated movements of proteins to understand the most fundamental movements towards channel opening. Covariance matrices were constructed using the backbone phi/psi trajectories, which contain all the information needed to reasonably describe the large synergistic motions of proteins. The free energy contour plots in Figure 4 were constructed for cTRPV1-oxi at 300 K. The span of PC1 and PC2 movement modes in cTRPV1-oxi is large, indicating substantial conformational rearrangements between the two states. The span of PC1 and PC2 movement patterns in the other three systems is not obvious. Among them, the relatively clear one is oTRPV1-red, whose PCA plot is somewhat similar to that of cTRPV1-oxi (Figure S6). These results are consistent with the results from the pore radius calculation. The cTRPV1-oxi system was then selected to elucidate the conformational transmission of TRPV1 activation.

### 2.4. Redox Inducing Motions of TRPV1 Structure

To investigate the expansion/contraction motions of the pore, the radius of gyration (Rg) of the upper/lower gates, the pore region, and the transmembrane helix were calculated (Figure S7). The results show that the upper gate stays comparably stable and contracts in all states investigated during the simulations, with Rg at around 1.45 Å. The lower gate was comparatively expansive, with Rg at about 2.1 Å. Thus, the motion of the upper gate is one of the most important determinants for ion or water penetration in the TRPV1 channel.

**Figure 5:**
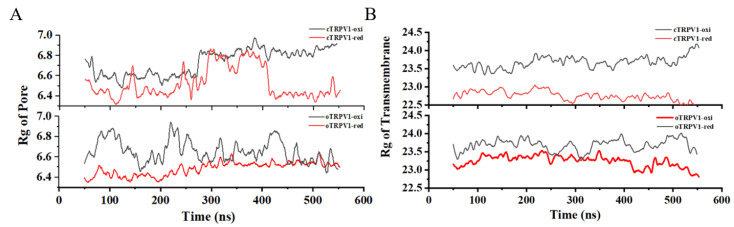
The Rg profiles, unit: Å. (**A**) the Rg of pore; (**B**) The Rg of transmembrane domain.

The oxidization-related expansion occurs in the pore and transmembrane helical regions (Figure 5A). Compared with the initial structures, the pore region expanded in cTRPV1-oxi and slightly contracted in cTRPV1-red. In this process, the pore Rg of cTRPV1-oxi is always greater than that of cTRPV1-red. The expanded pore is conducive to ion penetration. Therefore, cTRPV1-oxi has a larger pore radius than cTRPV1-red, which is consistent with the observation of pore radius changes. In addition, the Rg of the pore in oTRPV1-oxi is always bigger than that in oTRPV1-red. This is consistent with the results that show that the mean hole radius of oTRPV1-oxi is larger than that of oTRPV1-red. It is not surprising that the Rg of a pore is related to the pore size since the key determinant of the pore size is the upper gate, which is located in the pore region. However, there is an abnormal peak. In the pore radius curve on the oTRPV1-oxi system, which was not observed in the Rg of the pore curve. It suggests that there should be some other factors influencing the opening of the channel besides the Rg of the pore. 

To further investigate the conformation changes related to the pore, we calculated the Rg of the transmembrane domain (Figure 5B). For closed TRPV1, the transmembrane Rg profiles are clearly differentiated at the beginning of 50 ns. The oxidation state causes the transmembrane region to expand since the Rg values are obviously larger in the oxidation state than in the reduction state. In order of Rg from largest to smallest, the four systems are cTRPV1-oxi, oTRPV1-oxi, cTRPV1-red, and oTRPV1-red, and their order seems to be similar to the order of pore radius. However, no obvious linear correlation was observed between the pore radius curve and the Rg of the transmembrane curve. As we expected, the opening of the channel is probably the result of multi-variable synergy.

### 2.5. Pairwise Distance Analysis

**Figure 6:**
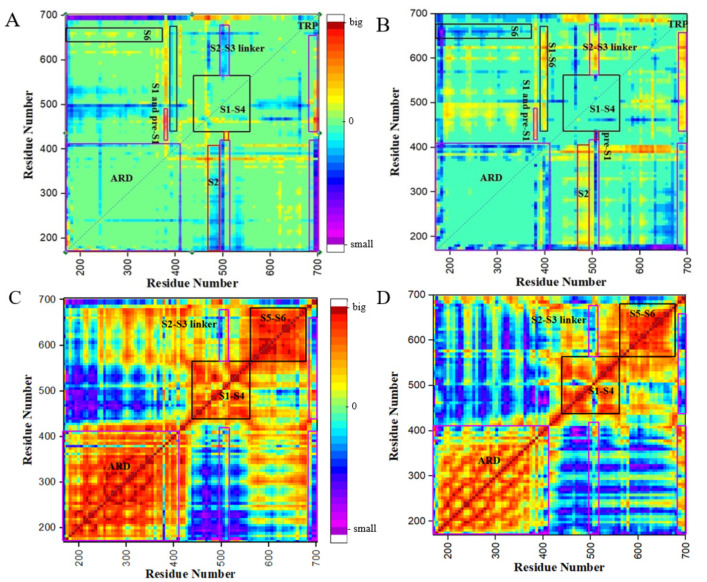
The pairwise distance matrix of cTRPV1 (**A**) and oTRPV1 (**B**) in different redox states and heat map of correlation coefficient of the distance matrix of cTRPV1 (**C**) and oTRPV1 (**D**).

To further complement the Rg analysis, we performed pairwise distance analysis on the 2000 snapshots from the aMD trajectories to monitor the relative domain motions in different redox states (Figure 6A,B). Considering the above observation that the channel is partially activated in the oxidized state and stabilized in the closed conformation in the reduced state, the pairwise distance variation may be closely related to the conformational changes of the channel. For the vast majority of residues, the pairwise distance changes are close to zero as the redox environment changes, suggesting that some local conformational changes modulate the conformational changes. Thus, investigation of the detailed conformational transfer is essential in elucidating the molecular basis of TRPV1-mediated redox signal transduction. 

The cysteine pairs C387 and C391 are located at the C-terminus of ARD, near the intra-subunit MPD linker. In the oxidized state, the C387–C391 disulfide bond moves away from pre-S1, which plays key roles in correct folding and channel assembly [43]. The MPD linker moves closer to S1–S6 in the oxidized state and moves away from S1–S6 in the reduced state. The movement is consistent with the high flexibility of the MPD linker revealed in the RMSF analysis (Figure 2) and may relate to the trend of the channel opening. The oxidized cysteine pair probably modulates the channel opening by regulating pre-S1 and the adjacent MPD linker. The connecting MPD, which is crucial for temperature sensing and heat activation [44], is probably involved in the activation of the TRPV1 channel. Therefore, oxidative stress-induced TRPV1 channel activation and thermal activation have some common features.

The S2–S3 linker is located at the binding site of capsaicin [45], a TRPV1 agonist, and is thus also important for TRPV1 activation. The whole S2–S3 linker moves closer to S5 and S6, and the N-terminus of the S2–S3 linker moves closer to ARD when the channel is partially activated. At the same time, the S2–S3 linker moves away from pre-S1. The reverse motion occurs when the channel is gradually closed. As the S2–S3 linker moves, the site connecting S2 and S3 moves closer or further away from the ARD. Thus, the S2–S3 linker is essential for the conformational changes and is associated with redox stress-related TRPV1 activation. This is consistent with previous proposals that the S2–S3 linker is associated with vanilloid and heat-activated pathways [25,45].

A big movement was observed on the TRP helix, whose N-terminal was not affected by the redox environment, while the C-terminus underwent obvious conformational changes in different redox states. This is consistent with previous reports that the TRP helix is implicated in the allosteric coupling between stimulus sensing and pore opening [6,26,46]. The pairwise residue distances between TRP and S1–S6 changed significantly in the two redox states. As the channel closes, short-range movement happens between the TRP helix and ARD. In addition, the distance between the TRP helix and the transmembrane helices gets longer in the oxidized state than in the reduced state. Since the TRP helix mediated the domain interaction between ARD and the S1–S6 transmembrane domain, the significant conformational changes revealed that the TRP helix is involved in the conformational change of the TRPV1 inter-domain. Combined with its unique localization, the TRP helix appears to be a molecular determinant of channel gate activation [23]. 

To further investigate the relationship between the motions, we performed a correlation coefficient analysis on the distance matrix. It is clear from Figure 6C,D that there are four regions: the ARD, S1–S4, S5–S6, and TRP helix, which are positively correlated for their intra-domain motions in different redox environments. It indicated that these domains are structurally stable within domains, and they each moved as a unit during conformation transfer. The motions of ARD and S1–S4 were always negatively correlated at different redox states. The motion of ARD was positively correlated with the motion of S5–S6 during partial activation of the channel and negatively correlated with the motion of S5–S6 during channel closure. The TRP motion showed a weak negative correlation with the motion of S5–S6 and ARD during pore opening and a weak positive correlation with pore closure. Thus, the motions of ARD, S1–S4, and S5–S6 are closely correlated. 

In summary, the pairwise residue distance heatmap showed that the apparent motions occurred in the MPD linker, the S2–S3 linker, and the TRP helix. In addition, the S4–S5 linker may also have conformational changes, leading to relative motion between S1–S4 and S5–S6. The movement of ARD, S1–S4, and S5–S6 domains is probably regulated mainly by conformational changes in the MPD linker and the S4–S5 linker. The regions involved in oxidative stress-induced TRPV1 activation overlapped with the regions involved in thermal activation and capsaicin activation.

### 2.6. Hydrogen Bond Analysis

Based on the pairwise distance analysis, we further investigated the details of the hydrogen bond interactions formed in the transmembrane domain and the juxtamembrane region. The hydrogen bonds during the simulation were counted in two different redox states, with a special focus on the following regions: S2–S3 linker, pore, S4–S5 linker, and the flexible ARD (Table S1). The main differences in hydrogen bond formation probabilities, >20% between the oxidized and reduced states, were recorded. Statistically, most of the H-bond formation or breakage occurred in the S2–S3 linker and pore region, and a minority of H-bond changes occurred in the S4–S5 linker and ARD. The regions with dynamic H-bonds were similar to those associated with thermal activation; however, the amino acid residues involved did not overlap [25]. Two of those H-bonds between the upper gate M645 and K640, T642 and Y667, are only present in the reduced state but not in the oxidized TRPV1. 

### 2.7. TRPV1 Channels Opening Mechanism

**Figure 7:**
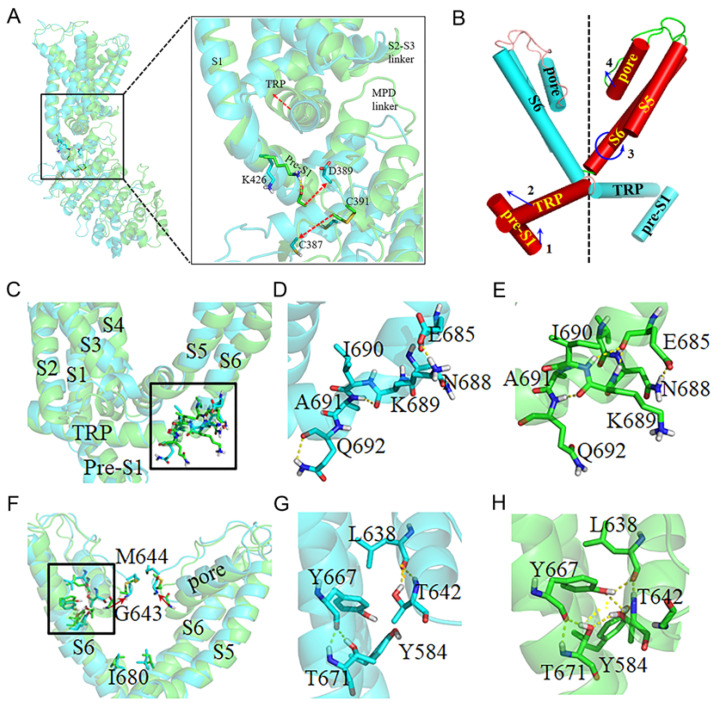
The opening mechanism of TRPV1 channel. The closed conformation was from cTRPV1-red system and colored cyan, the open conformation was from oTRPV1-oxi system and colored green. The key residues were labeled and shown in sticks. The yellow dashed line represents a hydrogen bond, and the red dashed arrow indicates the moving direction of the TRP as the channel opening. (**A**) The represented TRPV1 conformation were superimposed, and the key conformational changes around the cysteine pair were highlighted; (**B**) The conformational transmission was shown by cartoon, where the arrows represent the moving direction as TRPV1 activation; (**C**–**E**) The local conformational change and hydrogen bonds switch in the S6-TRP linker; (**F**–**H**) The key interaction and conformational comparison of pore helix.

To explore the molecular basis of channel opening, representative structures, representing the closed and open states of TRPV1 structures, were extracted and systematically investigated (Figure 7). 

Conformational analysis demonstrates that the intramembrane domain rotates flexibly around the TRP helix during molecular dynamic simulations. As a result, a significant structural change was observed at the C-terminus of the TRP helix based on the pairwise distance matrix. The disulfide bond between C387 and C391 limits the conformation and position of D389, which forms an abundance of H-bonds with the MPD linker (Figure 7A). In the reduced state, D389 is flexible and forms electrostatic and H-bond interactions with K426 of pre-S1. The favorable interaction allows pre-S1 to twist toward the cysteine pair. This observation is consistent with the pairwise distance analysis, where the distance between the cysteine pair and pre-S1 is longer in the oxidized state than in the reduced state. The conformational change of pre-S1 has a direct effect on the movement of the C-terminus of the TRP helix, which is surrounded by pre-S1, the MPD linker, and the S2–S3 linker. The movement of TRP induces the motions of the S2–S3 linker and MPD linker, which are observed in pairwise distance analysis. The conformational change of the MPD linker gives high flexibility to the ICD, which is beneficial for signal transduction. 

Combining the above RMSD and twist angle analysis, the motion of the TRP helix probably twists the connecting S6, while the well-structured S6-TRP linker fine-tunes the extent of its twist through the transfer of H-bonds. In the closed TRPV1, the NH of A691 forms an H-bond with the carbonyl oxygen of K689, and the acidic residue E685 forms an electrostatic interaction with the basic residue K689 (Figure 7C,D). In the partially activated state, the carbonyl oxygen of K689 forms an H-bond with the NH of Q692, which leads to a slight twist of S6 (Figure 7E). In addition, the carbonyl oxygen of E685 forms two H-bonds with the backbone NH of I690 and K689, which further leads to the twist of S6. The twist of S6 can further affect the conformation of the pore region. The H-bond transfer probably results from the motion of the TRP helix and is closely related to the activation of TRPV1. 

The pore helix is closely related to the pore opening. In the closed state, the hydroxyl group of the pore residue T642 forms H-bonds with T671, Y584, and Y667, the last of which is reduction-sensitive as mentioned above. The phenolic hydroxyl group of Y667 forms an H-bond with the carbonyl oxygen of L638. Additionally, the basic sidechain of K640 forms a reduction-sensitive H-bond with the main chain of M645, which points to the center of the pore. In the partially activated state, similar H-bonds were observed, except for the H-bond between K640 and M645 that broke. Without this H-bond, the main chain of M645 flipped, and its side chain moved upward and away from the hole center. As a result, the radius of the pore is enlarged, and the channel is gradually opened.

Combined with the above conformational analysis, we propose the hypothesis that the oxidation of the cysteine pair C387–C391 activates or partially activates TRPV1. The oxidized C387–C391 induces a conformational change in pre-S1, which causes it to move toward the TRP helix. Meanwhile, the pre-S1 acts as a lever, and its motion leverages the TRP helix to move upward (Figure 7B). As a whole, the connecting S6 moves away from the channel axis. In addition, the S6-TRP linker modulates the range of S6 movement by H-bond transfer, which also leads to the twist of S6. Along with the motion of S6, the pore helix slides upward along the inner surfaces of S5 and S6. Furthermore, the H-bond formed between the pore residues K640 and M645 is a close signal for TRPV1, and with the breakage of the H-bond, the channel opens. 

## 3. Materials and Methods

### 3.1. Homology Modeling of Human TRPV1 Structure

The homology modeling was performed using the Biologics module implemented in the Schrödinger suite 2021 (Schrödinger, LLC, New York, NY, USA, 2021) [47]. The primary sequence of human TRPV1 was obtained from the Uniprot database [48] (Uniprot ID: Q8NER1). BLAST was used to find homologous protein structures in the PDB database, using BLOSUM62 [49] for similarity analysis. 

In order to construct the model of closed TRPV1 (cTRPV1), the structures of rat TRPV1 in the closed state (PDB: 7RQU [33]) and the ankyrin repeat domain of human TRPV1 (PDB: 6L93 [50]), were retrieved from the Protein Data Bank and served as templates. The sequence alignment between the template and target was carried out using ClustalW2.1 [51]. After structure and sequence alignment, the coordinates of residues 112–360 were copied from 6L93, and residues 361–720 were built using 7RQU as the template. Finally, the top five TRPV1 tetramer models were built, arranged according to the arrangement of 7RQU [33]. The construction method of open-state human TRPV1 (oTRPV1) was similar to that of cTRPV1. The rat TRPV1 in open state (PDB: 7RQW [33]) and 6L93 [50] were used as the templates. The residue segments 112–360 of oTRPV1 were built based on 6L93, and residues 361–720 were modeled based on 7RQW. The human oTRPV1 tetramer was arranged using 7RQW as the template. During the model establishment, all non-template residues were minimized, and side chains were optimized. Finally, the top five models were generated using the knowledge-based method. 

All models were validated by Ramachandran Plot. The best two models, cTRPV1 and oTRPV1, were then chosen and pre-prepared by charge and hydrogen addition using the protein preparation wizard of Schrödinger, and the protonation states of the residues were predicted using PROPKA [52]. The disulfide bond between C387 and C391 was added manually (suffixed with -oxi) or not (suffixed with -red). Therefore, four systems, oxidized cTRPV1-oxi and oTRPV1-oxi and reduced cTRPV1-red and oTRPV1-red, were obtained. Each model was initially energy minimized for 500 steps using the steepest-descent (SD) method, followed by 4500 steps using the Polak-Ribier conjugate gradient (CG) method by using MacroModel software [47].

### 3.2. Molecular Dynamic Simulations

The four TRPV1 systems were further optimized using molecular dynamic simulations. The setup was done with the membrane builder function of the CHARMM-GUI server (http://www.charmm-gui.org accessed on 30 April 2023), and the hydrogen coordinates were preserved. Then, the protein was embedded in a pre-equilibrated 170 × 170 Å POPC (1-palmi-toyl,2-oleoyl-sn-glycero-3-phosphocholine) lipid bilayer membrane. For both oTRPV1 systems, a total of 695 (356 upper leaflet, 339 lower leaflet) POPC molecules were added using the replacement method, and for both cTRPV1 systems, a total of 698 (356 upper leaflet, 342 lower leaflet) POPC molecules were added. Then, each system was put in a water box of 170 × 170 × 150 Å^3^ with a 150 mM concentration of NaCl. The minimum water height on top and bottom of the system was 22.5 Å. 

The all-atom model was generated using the xleap module in Amber18 [53] on the basis of the optimized models. The TIP3P water model was used for solvent. The ff14SB [54] all-atom Amber force field was used for the protein, while the lipid14 [55] force field was applied for the lipid bilayer membrane. 

The minimization of each of the four systems is carried out successively using 10,000 steps of SD and 10,000 steps of CG minimization. The nonbonded forces (van der Waals and electrostatics) are evaluated within a cutoff distance of 10 Å. Then, the system was gradually heated to 303 K over 120 ps with a time step of 1 fs using the NVT ensemble. In this procedure, the solute was restrained with a 10 kcal·mol^−1^·Å^−2^ harmonic force constant. The equilibration is performed in 50 ns of dynamics with a time step of 2 fs in the NPT ensemble. The resulting system was used in subsequent accelerated molecular dynamic simulations (aMD). The aMD allows the possibility of boosting the whole potential with an extra boost to the torsions. It was performed with a time step of 10 fs in the NVT ensemble. Finally, triplicate accelerated MD simulations for either of the systems were conducted at 1 mpa and 303 K, and the production running time is about 500 ns.

### 3.3. Root Mean Square Fluctuation Analysis

The snapshots were extracted from the final 500 ns aMD trajectories every 100 ps. In addition, the snapshots were superimposed onto the first structure by the backbone of residues 432–713. The solvent molecules, ions, and membrane molecules were stripped. Then the root mean square deviation (RMSD) of the trajectories and the root mean square fluctuation (RMSF) of the residues were calculated using the cpptraj program. To monitor the relative flexibility of each residue in different models, we calculated the relative change rates of RMSF of two systems ($∆RMSF/\bar{RMSF}$), which are calculated by Formula (1).

(1) $$∆RMSF/\bar{RMSF}=2\times ({RMSF}_{1}-{RMSF}_{2})/({RMSF}_{1}+{RMSF}_{2})$$

where RMSF_1_ is the RMSF in one system and RMSF_2_ is the RMSF in the other one.

We calculated the $∆RMSF/\bar{RMSF}$ values of TRPV1 in different redox states by using the RMSF of oxidized TRPV1 (RMSF_TRPV1-oxi_) as RMSF_1_, and the RMSF of reduced TRPV1 (RMSF_TRPV1-red_) as RMSF_2_. The $∆RMSF/\bar{RMSF}$ values were also calculated for the models with the same redox state, where the RMSF of oTRPV1 (RMSF_oTRPV1_) was RMSF_1_ and the RMSF of cTRPV1 (RMSF_cTRPV1_) was RMSF_2_.

### 3.4. Rg Analysis of Inward/Outward Domain Motions

The cpptraj program was used to calculate the radius of gyration (Rg) over time for key regions, including the upper gate, lower gate, and pore region, based on the coordinates of heavy atoms. 

### 3.5. Radius of the Hole

A total of 2000 snapshots were extracted from the last 500 ns of aMD trajectories, and the radius of the channel of each snapshot was monitored using the HOLE 2.0 program [38]. The minimum radius was extracted and plotted as a curve by running time. The flow of water passing through the pore was computed using a Python script implemented with the MDAnalysis package [56].

### 3.6. Distance Matrix Analysis of Interdomain Motions

The same 2000 snapshots as mentioned above were used to calculate the average distance matrix of all residues in the same TRPV1 subunit based on the Cα atoms. The calculation was performed with the cpptraj program. Then the pairwise distance difference between TRPV1 in the oxidized state and TRPV1 in the reduced state was calculated to quantify the domain motion difference in different redox states. 

### 3.7. Twist Angle Calculations

The S6 twist angle of each subunit was determined by measuring angles between the channel axis, which is also named the Z axis, and the projected principal axis of each S6 onto the plane perpendicular to the radial vector r, which is perpendicular to the Z axis. The principal axis of each S6 was obtained by three-dimensional least-squares fitting of the Cartesian coordinates of the Cα atoms. The S6 helix includes residues from A658 to Q692. 

### 3.8. Hydrogen Bond Analysis of Dynamic Interdomain Interactions

A hydrogen bond (HB) is defined as being between an acceptor atom A, a donor hydrogen atom H, and a donor heavy atom D. The geometric criteria to identify a HB here are: the distance between A and D is less than 3 Å, the A-H-D angle is greater than 135°.

## 4. Conclusions

TRPV1 can be directly activated by oxidants through cysteine modifications and is involved in redox sensing. By analyzing the structure of TRPV1, we noticed that the cysteine pairs C386–C390 are spatially close to each other. Their side chains may be oxidized to form a disulfide bond, or they may exist in the reduced state as free thiol groups. Through multiple template-based homology modeling approaches, we have established the structures of human TRPV1 in open and closed states. We investigated for the first time the mechanism of activation of human TRPV1 when the cysteine pair is in the oxidized or reduced state by using the accelerated molecular dynamics method. The oxidized cysteine pair C387–C391, forming a disulfide bond, was used to simulate the oxidizing environment, and the free C387 and C391 represented the reducing environment. The simulation results showed that the oxidizing environment partially activated TRPV1 by structural rearrangement of the closed conformation, while the reducing environment inactivated TRPV1 mainly by stabilizing the closed conformation. Regions including Pre-S1, TRP helix, S6, and pore helix participated in the pore opening through H-bond transfer. After a detailed conformational analysis, we proposed a TRPV1 activation mechanism. That is, pre-S1 acts as a lever to pry the TRP helix upward, thus moving S6 away from the central axis, and the S6-TRP linker can also modulate the motion of S6, which further influences the movement of the pore region. Finally, the motion of the pore and the breakage of an H-bond between K640 and M645 partially activate TRPV1. The discovery of key residues or segments provided a structural basis to guide future functional studies of TRPV1. The results provided new insights into the mechanisms of TRPV1 activation by the redox states of cysteine pairs C387–C391 and helped to re-evaluate the redox sensitivity of TRPV1, which is crucial for making significant advances in the treatment of human diseases.

## Data Availability

The authors state that all the data necessary to replicate the results is presented in the manuscript. Relevant parts of the code can be shared upon contacting the corresponding author.

## Supplementary Materials

The supporting information can be downloaded at: https://www.mdpi.com/article/10.3390/ijms24119553/s1.
