# Supplementary material for: Molecular Dynamic Simulations Reveal the Activation Mechanisms of Oxidation-Induced TRPV1
Source: Int J Mol Sci. 2023 May 31;24(11):9553. doi: 10.3390/ijms24119553 (PMC10253787; doi:10.3390/ijms24119553)
Supplement: Supplementary file 1 [file ijms-24-09553-s001.zip › ijms-2403231-supplementary.pdf]

# Molecular dynamics reveal the activation mechanisms of oxidation-induced TRPV1

Yanyan Chu[1,2,*], Huanhuan Zhang[1], Mengke Yang[1], Rilei Yu[1,2,*]

[1]*Marine Biomedical Research Institute of Qingdao, Ocean University of China, 5 Yushan Road, Qingdao 266003, China*

[2]*Innovation Platform of Marine Drug Screening & Evaluation, Pilot National Laboratory for Marine Science and Technology (Qingdao), Qingdao, 266100, Shandong, China.*

*Corresponding Author E-mail: Yanyan Chu: chuyanyan@ouc.edu.cn

Construction of the complex of hTRPV1 and RhTx

The structure of hTRPV1 from residue L112 to A720 was established by truncating the residues D605-S630 because the corresponding residues were unsolved in all structures of rTRPV1. The sequence alignment shows that hTRPV1 is highly homologous with rTRPV1, with sequence identity of 90.8% and sequence similarity of 96.6% (Figure S1). By using multiple templates and homotetramer homology modeling, we obtained the structures of hTRPV1 in two states: the closed state (cTRPV1) and the open state (oTRPV1). The generated models were evaluated using Ramachandran Plot (Figure S2). According to the evaluation, the best models of cTRPV1 and oTRPV1 were chosen for further investigation. The plot showed that most of the residues located in the favored or allowed regions. The residues in the disallowed region were checked and modified.

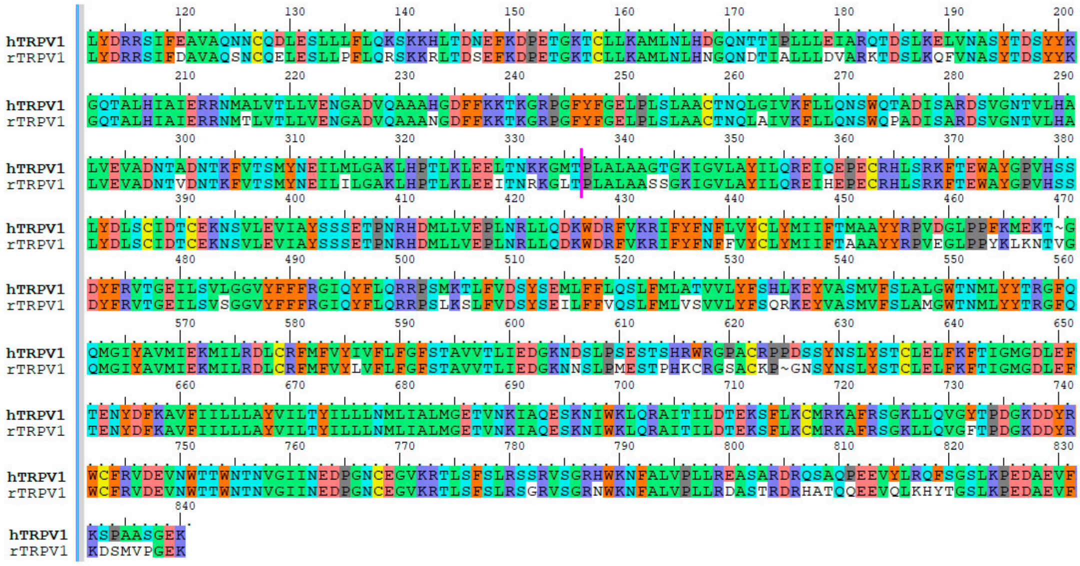

**Figure S1.** The sequence alignment of hTRPV1 (Q8NER1) with rTRPV1 (O35433).

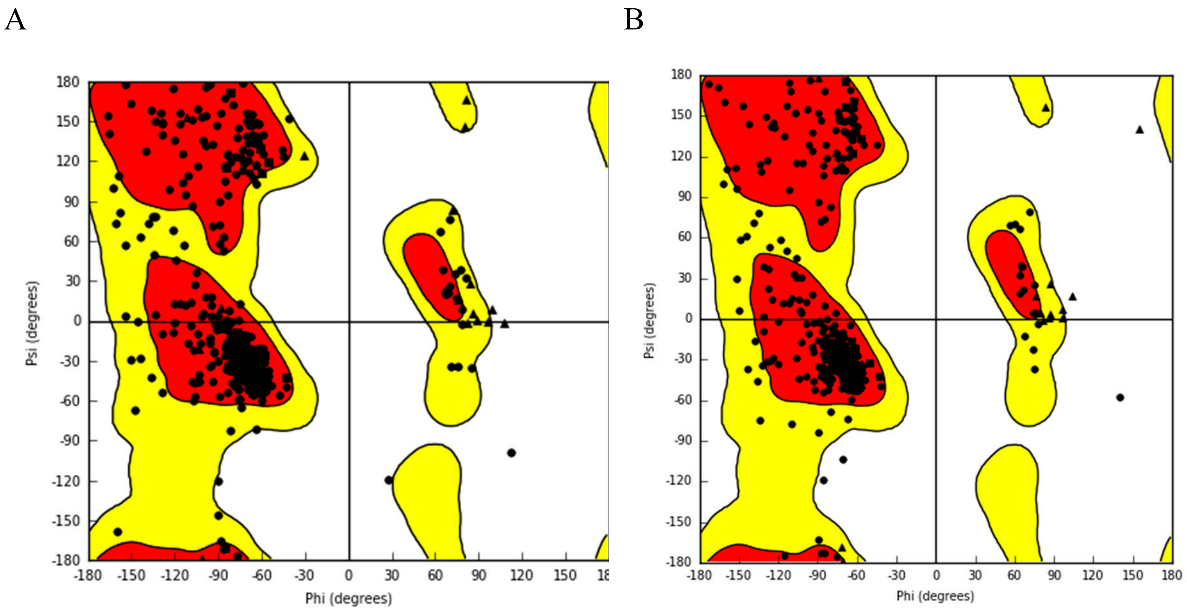

**Figure S2.** The Ramachandran Plots of oTRPV1 (A) and cTRPV1 (B).

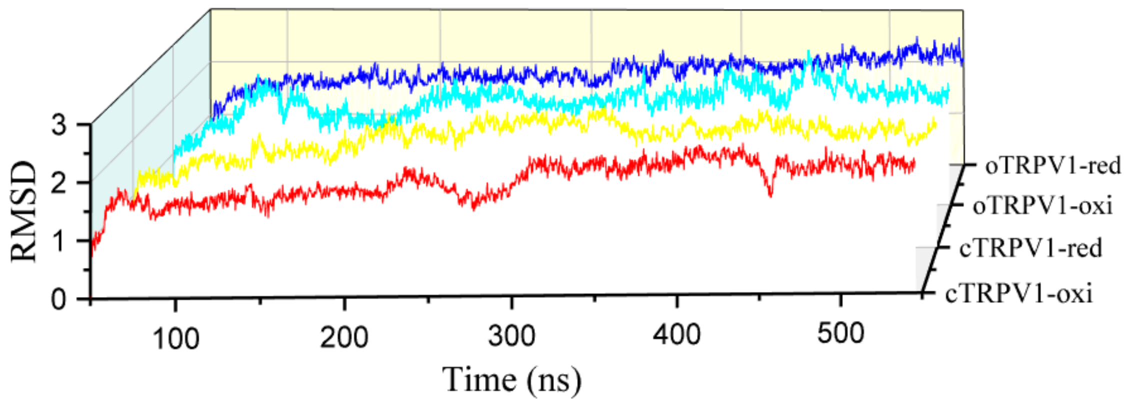


**Figure S3.** The RMSD profiles of TRPV1 transmembrane domain for representative MD trajectories for each of the four systems.

A.

B.

C.

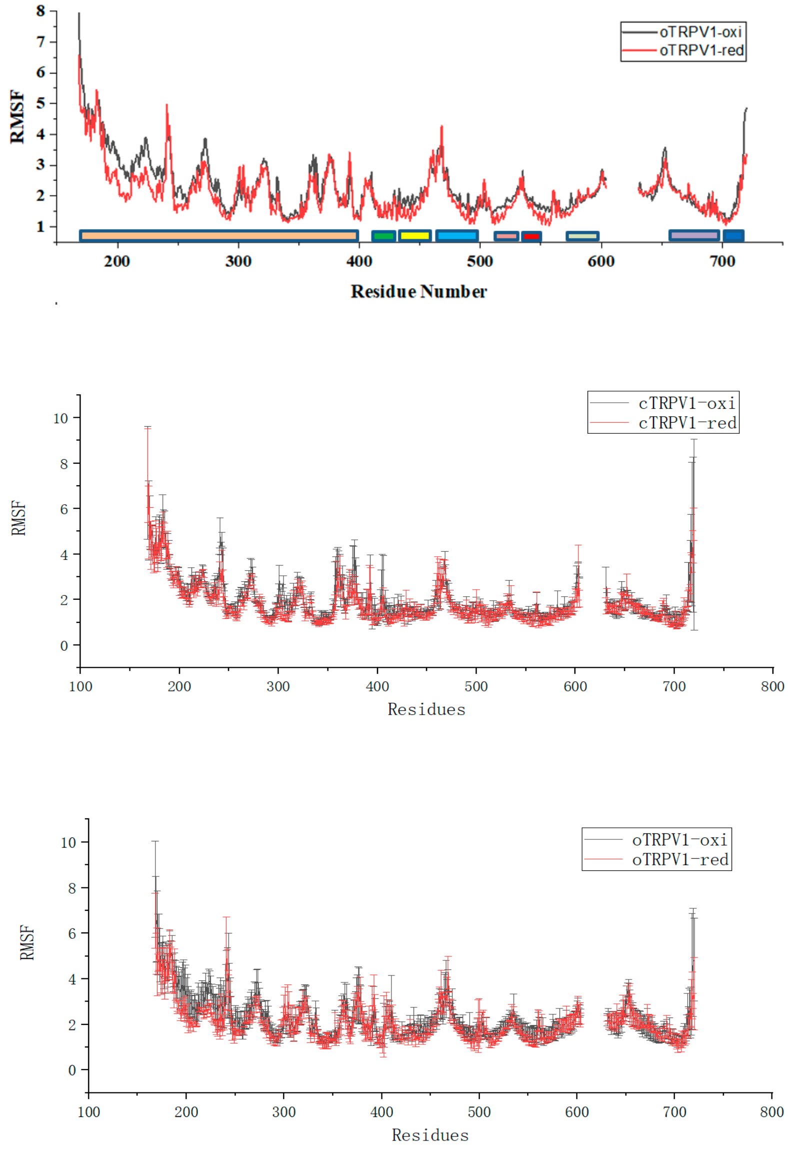

**Figure S4. The RMSF profiles. (A)** The RMSF values of each residue in oTRPV1-oxi (black line) and oTRPV1-red (red line) systems; (B) The mean RMSF values of each residue in different monomers of cTRPV1-oxi (in black line) and cTRPV1-red (in red line), with standard deviations indicated by error bars in the figure; (C) The mean RMSF values of each residue in different monomers of oTRPV1-oxi (in black line) and oTRPV1-red (in red line), with standard deviations indicated by error bars.

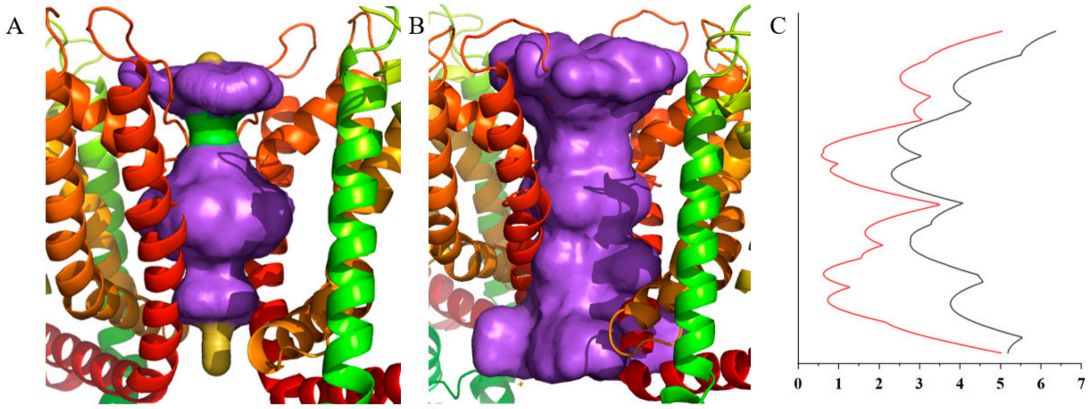


**Figure S5.** The pore of closed TRPV1 (A), open TRPV1 (B). (C) The pore radius of the closed (red) and open TRPV1 (black).

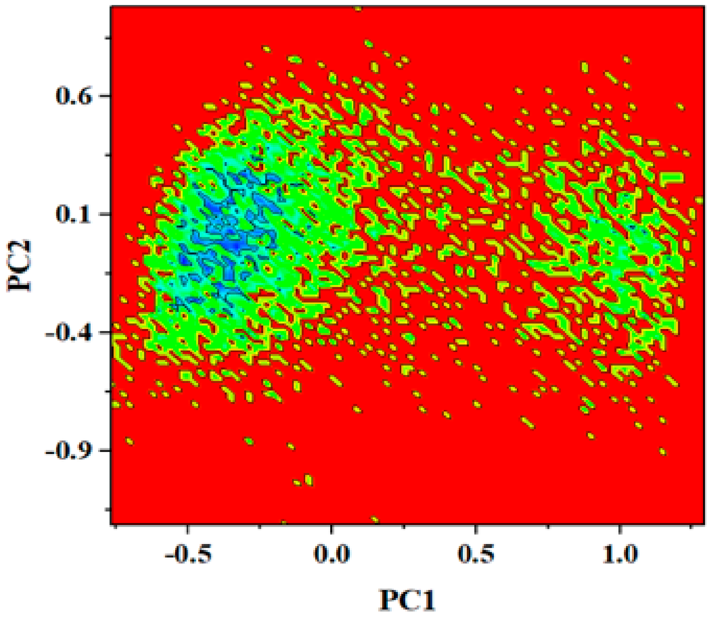


**Figure S6.** The principal component (PC) analysis of oTRPV1-red. From blue to red indicates the free energy from the lowest to the highest.

A

B

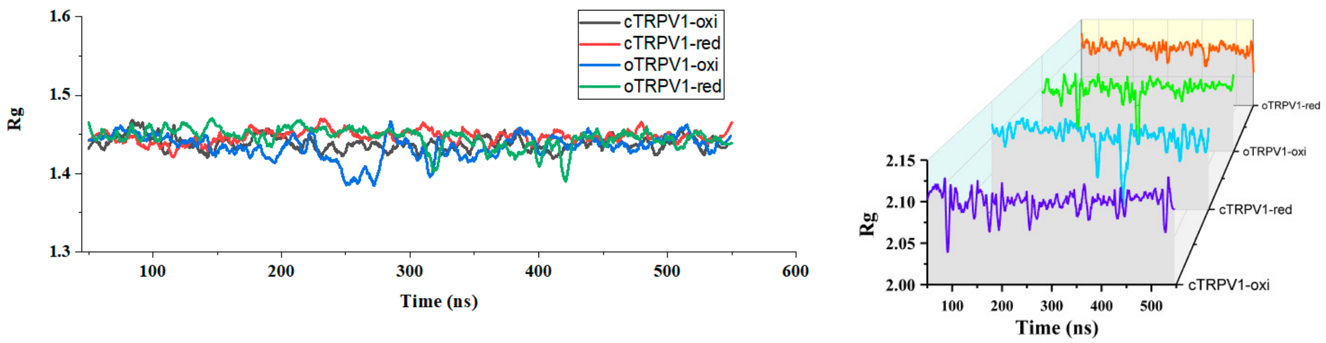


**Figure S7.** The radius of gyration of upper gate (A) and lower gate (B).

## Table S1

**Table S1.** The hydrogen bond forming residues and their occupancies.

| HB-Forming Residue Pair and Occupancy | | Oxi | Red | Residue location |
|---|---|---|---|---|
| D509 | R499 | 1.34 | 0.60 | S2-S3 linker |
| F496 | R500 | 0.72 | 0.33 | S2-S3 linker |
| S510 | M514 | 0.53 | 0.20 | S2-S3 linker |
| Y495 | R499 | 0.17 | - | S2-S3 linker |
| Y495@O | R499@H | 0.45 | 0.60 | S2-S3 linker |
| M503 | F507 | - | 0.22 | S2-S3 linker |
| T505 | S510 | 0.16 | 0.44 | S2-S3 linker |
| Q498 | W402 | 0 | 0.27 | S2-S3 linker-ARD |
| N604 | S633 | 0.95 | 0.38 | pore |
| N653 | G602 | 0.22 | 0 | pore |
| Y632 | L636 | 0.36 | 0.21 | pore |
| F656 | F660 | 0.27 | 0.11 | pore |
| F639 | T642 | 0.27 | 0.13 | pore |
| L636 | K640 | 0.29 | 0.45 | pore |
| F639 | I643 | 0.11 | 0.28 | pore |
| E637 | F641 | 0.18 | 0.38 | pore |
| T505 | D509 | 0.34 | 0.55 | pore |
| K640 | M645 | 0 | 0.56 | pore |

| | | | | |
|---|---|---|---|---|
| L638 | Y667 | 0.45 | 0.30 | pore-S6 |
| T642 | Y667 | 0 | 0.34 | pore-S6 |
| Y584 | T642 | 0.32 | 0.01 | Pore-S5 |
| F559 | G563 | 0.47 | 0.16 | S4-S5 linker |
